# Supplementary material for: Identification of photoperiod-induced specific miRNAs in the adrenal glands of Sunite sheep (Ovis aries)
Source: Front Vet Sci. 2022 Jul 22;9:888207. doi: 10.3389/fvets.2022.888207 (PMC9354845; doi:10.3389/fvets.2022.888207)
Supplement: Supplementary file 1 [file Data_Sheet_1.ZIP › Supplementary file/miRDeep2 file.zip!/miRDeep2 file/Novel_Structure/LP42A3/Novel_365.pdf]

Provisional ID	:	NC_019461.2_7497
Score total	:	0
Score for star read(s)	:	-1.3
Score for read counts	:	-1.8
Score for mfe	:	1.7
Score for randfold	:	1.6
Score for cons. seed	:	
Total read count	:	8
Mature read count	:	8
Loop read count	:	0
Star read count	:	0

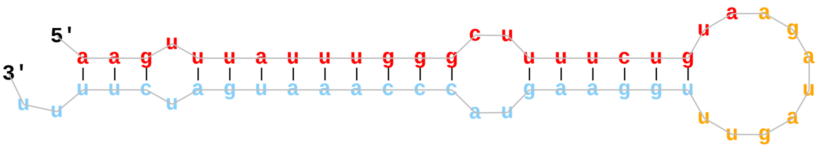

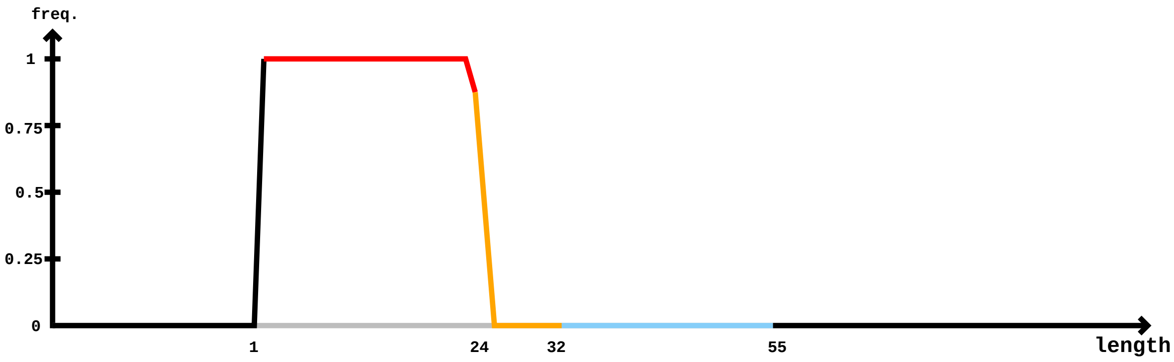


```
                    Mature                    Star
5'- aauguauuugguuggccaaaaaguuuauuugggcuuuucuguaagauaguuuggaaguacccaaaugaucuuuuuggucaacccaguauuugaagucaaggaagaugguggc   -3'   exp
    .........(((((((((((((.(((((((((..((((((..........))))))..))))))))).)))))))))))))((.(((((...........))))).))..   reads   mm   sample
    ....................aaguuuauuugggcuuuucugu......................................................................   1       0    seq
    ....................aaguuCauuugggcuuuucugua.....................................................................   7       1    seq
```
